# SAM-X: sorting algorithm for musculoskeletal x-ray radiography

**DOI:** 10.1007/s00330-022-09184-6

**Published:** 2022-10-29

**Authors:** Florian Hinterwimmer, Sarah Consalvo, Nikolas Wilhelm, Fritz Seidl, Rainer H. H. Burgkart, Rüdiger von Eisenhart-Rothe, Daniel Rueckert, Jan Neumann

**Affiliations:** 1 Department of Orthopaedics and Sports Orthopaedics, Klinikum rechts der Isar, Technical University of Munich, Ismaninger Str. 25, 81675 Munich, Germany; 2 Institute for AI and Informatics in Medicine, Technical University of Munich, Munich, Germany; 3 Department of Trauma Surgery, Klinikum rechts der Isar, Technical University of Munich, Munich, Germany; 4 Department of Diagnostic and Interventional Radiology, Klinikum rechts der Isar, Technical University of Munich, Munich, Germany

**Keywords:** Artificial intelligence, Deep learning, X-ray, Musculoskeletal diseases, Workflow

## Abstract

**Objective:**

To develop a two-phased deep learning sorting algorithm for post-X-ray image acquisition in order to facilitate large musculoskeletal image datasets according to their anatomical entity.

**Methods:**

In total, 42,608 unstructured and pseudonymized radiographs were retrieved from the PACS of a musculoskeletal tumor center. In phase 1, imaging data were sorted into 1000 clusters by a self-supervised model. A human-in-the-loop radiologist assigned weak, semantic labels to all clusters and clusters with the same label were merged. Three hundred thirty-two non-musculoskeletal clusters were discarded. In phase 2, the initial model was modified by “injecting” the identified labels into the self-supervised model to train a classifier. To provide statistical significance, data split and cross-validation were applied. The hold-out test set consisted of 50% external data. To gain insight into the model’s predictions, Grad-CAMs were calculated.

**Results:**

The self-supervised clustering resulted in a high normalized mutual information of 0.930. The expert radiologist identified 28 musculoskeletal clusters. The modified model achieved a classification accuracy of 96.2% and 96.6% for validation and hold-out test data for predicting the top class, respectively. When considering the top two predicted class labels, an accuracy of 99.7% and 99.6% was accomplished. Grad-CAMs as well as final cluster results underlined the robustness of the proposed method by showing that it focused on similar image regions a human would have considered for categorizing images.

**Conclusion:**

For efficient dataset building, we propose an accurate deep learning sorting algorithm for classifying radiographs according to their anatomical entity in the assessment of musculoskeletal diseases.

**Key Points:**

• *Classification of large radiograph datasets according to their anatomical entity*.

• *Paramount importance of structuring vast amounts of retrospective data for modern deep learning applications*.

• *Optimization of the radiological workflow and increase in efficiency as well as decrease of time-consuming tasks for radiologists through deep learning*.

## Introduction

Musculoskeletal diseases present a daily and also a global challenge for today’s healthcare system with far-reaching economic burdens to society and consequences to each individual who is affected by this disease. Finally, they result in pain and restriction of motion and interfering with the individuals’ quality of life [1]. Presenting a diverse group with respect to their pathophysiology, most conditions are, at least in part, classified according to the anatomical entity in which they are located. Hence, anatomy is being a crucial organizing principle for such diseases [2]. Accompanied by the ever-ongoing process of improving medical imaging with rapid changes and innovation [3], the recent dawning era of artificial intelligence (AI) and artificial neural networks will potentially lead to an increase of radiology exams. This will be especially true for medical imaging since it presents a cornerstone in the daily clinical routine and the workflow of shared care of patients with musculoskeletal diseases. Along the journey of new AI technologies, a variety of medical applications related to medical imaging has been noticed with the majority focusing on a head-to-head comparison of AI with humans [4]. However, modern imaging is more likely to involve human-in-the-loop setups, where humans actively collaborate with AI systems and provide oversight. When facing an increase of image data, the complexity of the radiology imaging workflow and corresponding amount of data promotes the need for possibilities to understand and use radiology data for gaining new knowledge and insights [5]. The complexity of medical imaging technologies implements the challenge for radiologists and neural networks to capture all details of each dataset, potentially reversing the hoped-for effect of reducing medical costs and time consumption when combining the workflow of human-AI collaboration. In order to avoid trade-offs to manage complex datasets in an active collaboration of humans with AI systems [6], pre-sorting algorithms according to the anatomical entity can be helpful to categorize large amount of image data, thus resulting in a more effective data analysis.

Multiple supervised, unsupervised, as well as self-supervised models have emerged [7–11] over the past years. These models could be utilized for sorting data. However, supervised learning requires a significant amount of annotated data and therefore demands for a substantial amount of time of a domain expert [12, 13]. In contrast, unsupervised and self-supervised models eliminate the need for time-consuming annotations, but clustering data follows mathematical rules such as similarity measures and consequently does not necessarily cluster data according to specific needs. In this study, we focus on utilization and modification of an established self-supervised model to categorize data effectively and efficiently according to specific requirements, while still keeping the demand of human interaction at a minimum. DeepCluster [8] demonstrates a self-supervising approach for learning image representation. The model iteratively groups features using a standard k-means clustering algorithm and uses the subsequent labels as supervision to update the weights of the network.

Therefore, in the present study, we propose a sorting algorithm for musculoskeletal X-ray radiography (SAM-X), a novel framework to support automatic classification and reasoning in the context of large image datasets.

## Materials and methods

### Dataset

The local institutional review and ethics board approved this retrospective study (N°48/20S). The study was performed in accordance with national and international guidelines. Informed consent was waived for this retrospective and anonymized study.

In total, 42,608 unstructured and pseudonymized radiographs were retrieved from the local Picture Archiving and Communication System (PACS) from a musculoskeletal tumor center (Klinikum rechts der Isar, Technical University of Munich) with sarcoma-associated ICD codes in DICOM format. The image data were collected over the past 25 years and contained heterogeneous data quality, resolution, and external images (~20%). Metadata such as DICOM header information or diagnoses are not yet validated and therefore not yet available.

**Fig. 1:**
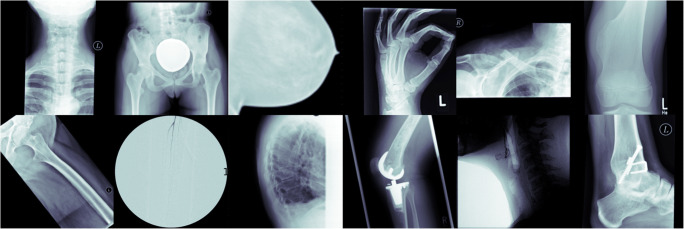
Exemplary data sample showing various anatomical entities from the initial unstructured data collective

All radiographs have been obtained through standard radiography techniques according to the body part imaged and in accordance with the radiographic manual procedures of our institution. Based on the aforementioned sarcoma-associated ICD code selection, data were acquired on potentially various points in time of the respective therapy status. Due to the various radiographic appearances and ubiquitous locations of sarcomas, our dataset includes a variety of all body parts, including potential anatomic variations, prosthetic devices, and medical implants. Figure 1 demonstrates examples from the dataset.

### Statistical analysis

The result of the initial clustering (phase 1) was assessed through a normalized mutual information (NMI) metric. NMI is a variant of a measure commonly used in information theory, called mutual information. Mutual information indicates the “amount of information” that can be extracted from one distribution with respect to a second one. The results of sorting the images in the classification task were measured with an accuracy score calculating the amount of correctly assigned images with respect to all images from the hold-out test set. Additionally, the accuracy for considering the two most probable prediction labels was computed. Since meta-information is not yet available, no distribution analysis of sex, gender, diagnoses, etc. was conducted.

### Model training

Model training and inference were conducted on a DGX Station A100 with four 80-GB graphical processing units (Nvidia Corporation, Santa Clara), 64 2.25-GHz cores, and 512-GB DDR4 system memory running on a Linux/Ubuntu 20.04 distribution (Canonical). Preprocessing and model implementation were performed in Python 3.9.6 (https://www.python.org/) using PyTorch 1.10.2 and cuda toolkit 11.3 (https://pytorch.org/). The trained classification model will be available on GitHub (https://github.com/) upon publication.

### Algorithm

**Fig. 2:**
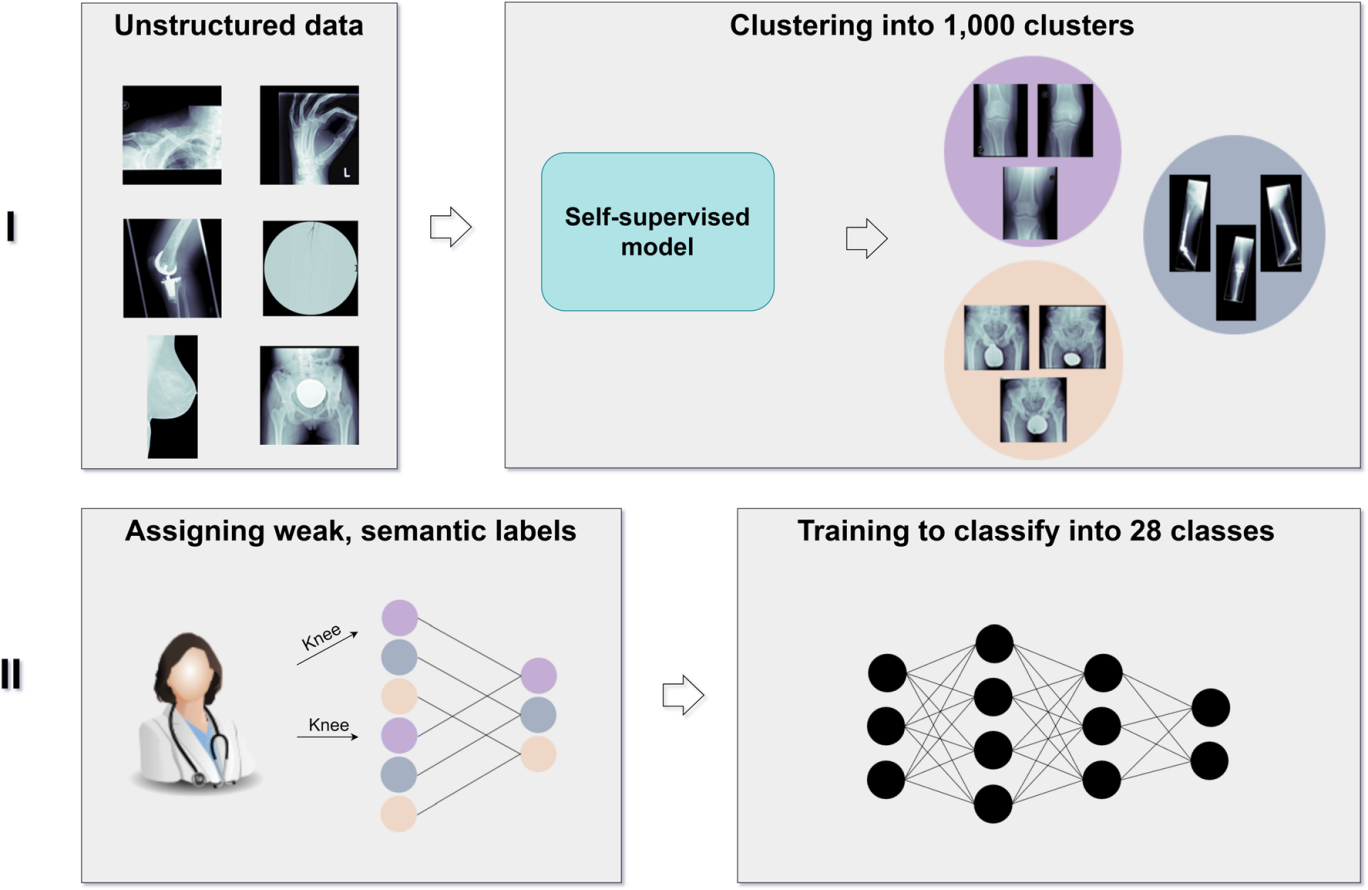
Illustration of the presented framework in two phases: clustering data with a self-supervised model and training a network with human-annotated clusters

A two-phase deep learning framework was developed consisting of a self-supervised model, human interaction through weak, semantic label assignment, and implementation of a supervised learning task for final training (Fig. 2).

In phase 1, the 42,608 unstructured and pseudonymized X-ray images from a musculoskeletal research storage were clustered into 1000 clusters by application of DeepCluster [8]. Following, a senior radiologist identified several musculoskeletal labels by screening the results from phase 1. Each of the 1000 clusters was either assigned a class label or discarded, since the images were not applicable for the task at hand (e.g., not a musculoskeletal X-ray such as upper gastrointestinal series/barium swallow, mammography). Clusters with the same class label were merged, so consequently “weak” (noisy) classes emerged. In phase 2, a convolutional neural network was trained on the emerged classes as weak, semantic labels: to accomplish an optimal training and update of the network’s weights, the created labels were “injected” into the same self-supervised model. The auxiliary labels for each training iteration from k-means became obsolete, since we provided the labels. It became a supervised task and the network’s weights were trained with respect to our classification requirements. In order to provide statistical significance and avoid cross-contamination, the data was split into training, validation, and hold-out test sets with a respective split ratio of 6-2-2, hence a 5-fold cross-validation was implemented. Half of the hold-out test set consisted of external imaging data to provide an independent and unbiased test set and increase significance of the results.

### Plausibility

To add plausibility and additional insight into the AI model, Grad-CAMs were implemented in the final inference step [14]. Grad-CAMs utilize the gradient information from the last convolutional layer of a deep learning network to understand specific neurons and their impact for decision-making. The result is a colored heat map, which is co-registered to the original input image and indicates where the algorithm found relevant information for the task at hand. This technique was applied to acquire a better understanding where the algorithm detects relevant information. To provide a higher expressiveness, the Grad-CAM results were averaged from the 5-fold cross-validation.

## Results

In phase 1, an NMI of 0.930 in clustering the entire dataset was reached. Subsequently, a senior radiologist identified the following 28 main musculoskeletal classes: abdomen, ankle, calcaneus, cervical spine, clavicula, elbow, femur, finger, foot, forearm, hand, hip, humerus, knee, lower leg, lumbar spine, paranasal sinus, patella, pelvis, ribs, sacrum, shoulder, skull, spine, thoracic spine, thorax, whole leg (standing), and wrist. In total, 13,175 non-musculoskeletal images from three hundred thirty-two clusters were discarded and a “musculoskeletal subset” of 29,433 images remained for further training. Table 1 shows the final classes with the number of images per class and percentage share with respect to the musculoskeletal subset.

**Table 1:** Final classes with the respective number of image samples and percentage share

Distribution of 28 classes		
Class	Number of images	in %
Abdomen	495	1.7%
Ankle	1033	3.5%
Calcaneus	177	0.6%
Cervical spine	3521	12.0%
Clavicula	100	0.3%
Elbow	186	0.6%
Femur	4270	14.5%
Finger	288	1.0%
Foot	904	3.1%
Forearm	192	0.7%
Hand	345	1.2%
Hip	1529	5.2%
Humerus	811	2.8%
Knee	3815	13.0%
Lower leg	1406	4.8%
Lumbar spine	564	1.9%
Paranasal sinuses	257	0.9%
Patella	269	0.9%
Pelvis	2134	7.3%
Ribs	88	0.3%
Sacrum	96	0.3%
Shoulder	1577	5.4%
Skull	79	0.3%
Spine	821	2.8%
Thoracic spine	814	2.8%
Thorax	3209	10.9%
Whole leg	212	0.7%
Wrist	241	0.8%
**Total**	**29,433**	**100**.**0%**

**Fig. 3:**
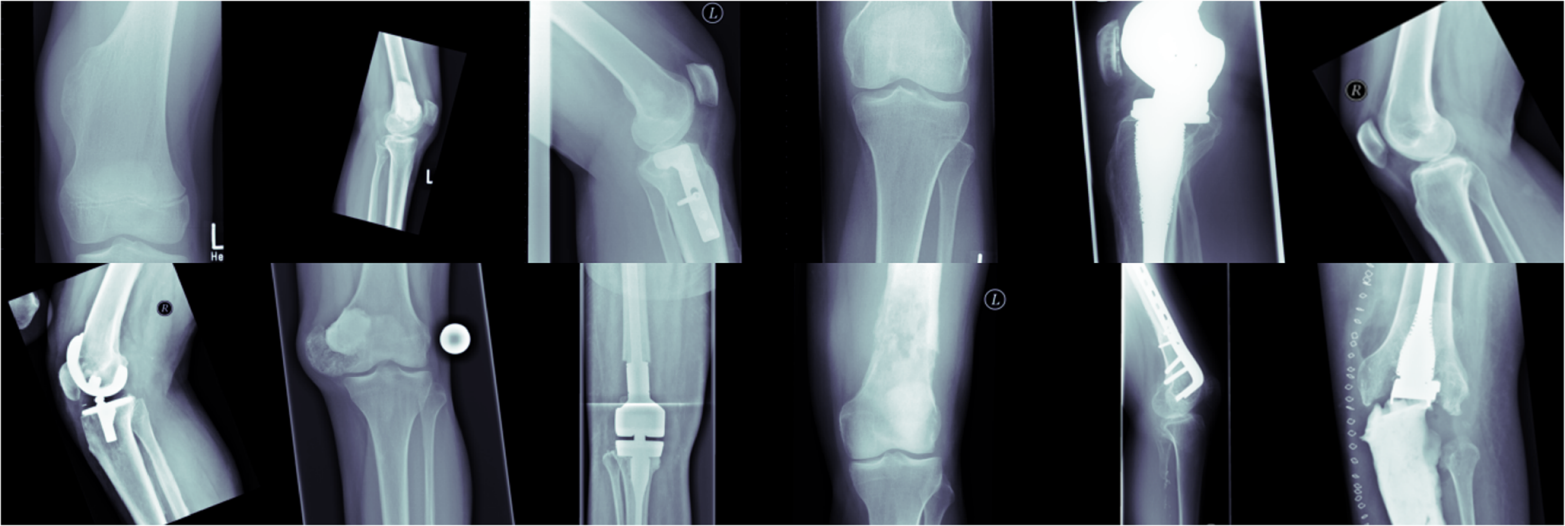
Illustration of the final *knee* class showing the correct anatomical entity (knee) despite underlying heterogeneous pathologies

In phase 2, a cross-validated classification accuracy of 96.2% for validation and 96.6% for hold-out test data was accomplished, when only considering the class with the highest prediction probability. When considering the top two predicted class labels, an accuracy of 99.7% and 99.6% for validation and testing was reached, respectively. Figure 3 shows examples of the final predictions of the *knee* cluster.

**Fig. 4:**
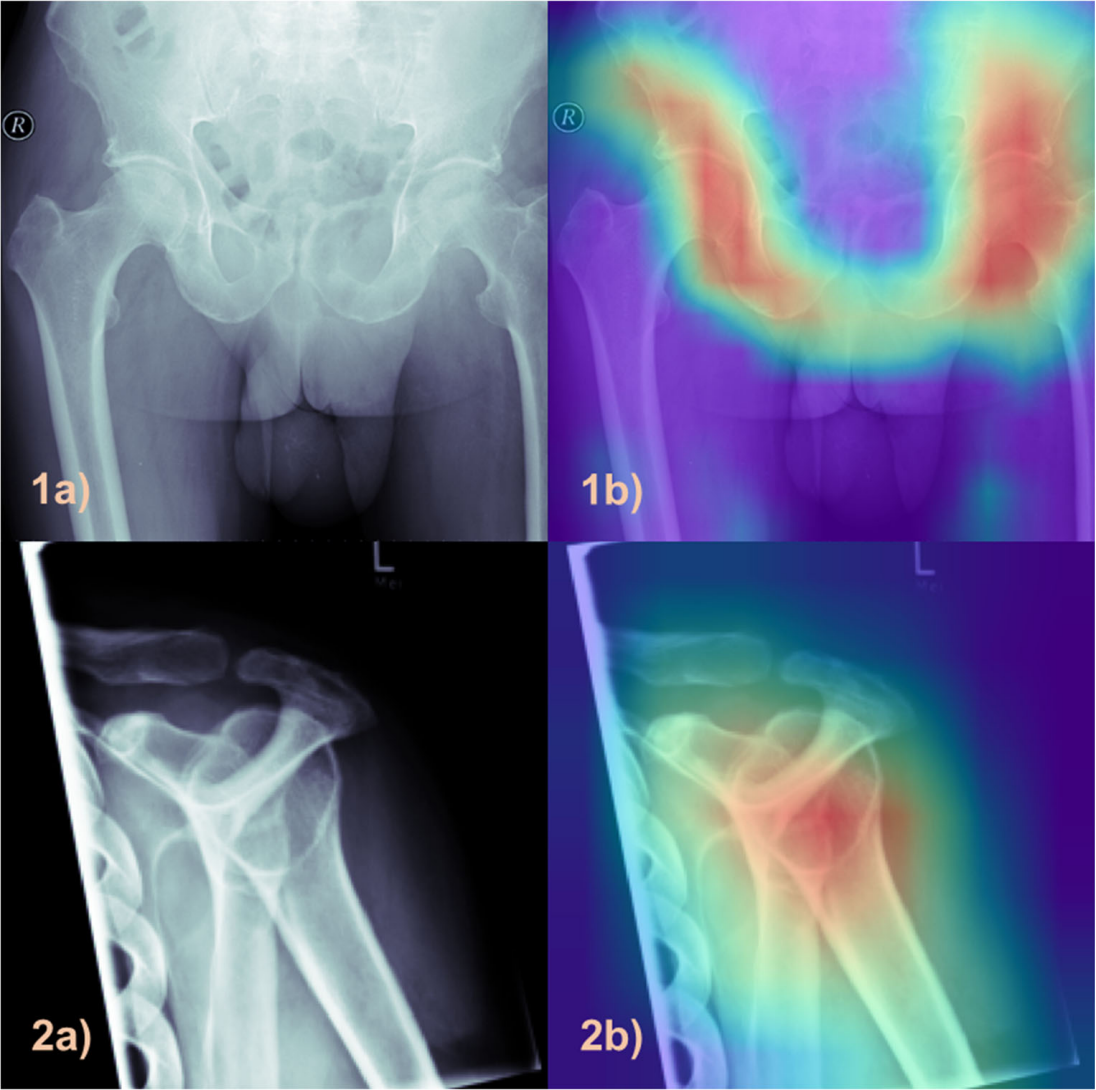
Grad-CAM examples from the classes pelvis (1a and 1b) and shoulder (2a and 2b) displaying the pixel areas, which were relevant for the algorithm to predict the according classes

Furthermore, Fig. 4 displays the results of the cross-validated Grad-CAMs for the classes *pelvis* (1a, 1b) and *shoulder* (2b, 2b). Purple pixels indicate that the algorithm did not find any relevant information in these pixels in contrast to red pixels, where most relevant information was detected.

## Discussion

In the present study, we demonstrate a sorting algorithm for musculoskeletal X-ray radiographs (SAM-X) to categorize images according to their anatomical entity. Based on a two-phase deep learning framework, 42,608 unstructured and pseudonymized radiographs have been categorized into a total of 28 anatomical regions. Cross-validated accuracy of 96.2% for validation and 96.6% for hold-out test data indicated a high accuracy and excellent reliability. The aforementioned final predictions of the knee cluster demonstrate that even very different pathologies and appearances of knees were correctly identified and categorized. Further, the calculated Grad-CAMs display the algorithms focus for predicting a specific class and help to unravel the black-box nature of DL methodology. These results underline the plausibility and robustness of the predictions: the algorithm primarily focused on anatomical regions, which are significant for the respective class and would also have been used by radiologists to determine the class.

Today, the emerging use of medical AI systems and neural networks presents an important, yet “still in the making” opportunity for our daily clinical routine and research. Radiology has always been at the front line of evolution in medical imaging since the introduction of digital imaging systems [15], teleradiology [16], computer-aided diagnosis tools [17], AI systems, and machine learning methods have emerged in the age of digitization. Ultimately, this has led to a significant increase of radiology examinations [17, 18]. Despite the broad use of previous research projects to analyze AI and humans in a head-to-head comparison [4, 9], promoting a potential level of lacking trustworthiness in these new technologies, future use is more likely practicing the human-in-the-loop setup used in our study as well, providing the possibility of humans actively collaborating with AI systems. Due to the complexity of clinical radiology examinations, accompanied by the amount of data linked to each examination, radiological daily operation may become inefficient, requiring tools to improve daily workflow and productivity [19]. Even more, with respect to image research, the most time-consuming part will become dataset building, potentially being the bottleneck [7] between data collection and creation of structured vs. unstructured data. Also, with respect to musculoskeletal disorders, radiologists, clinicians, and researchers face a diverse group of underlying pathologies which, in the setting of the aforementioned increasing number of radiology examinations and large image datasets, may benefit from pre-sorting algorithms to maintain order and effectivity. Although the aforementioned technologies have emerged, up to date, radiography still plays an essential and fundamental role for diagnosing, differentiating, and assessing the onset as well as progression of various musculoskeletal diseases [20, 24].

Mainly to harmonize occupational exposures and to flourish study comparison in meta-analyses for the use in occupational healthcare, surveillance, or research [25], the principle of categorizing musculoskeletal disease has already been widely used in the setting of identifying potential etiological or work-related factors that may lead to the onset or worsening of musculoskeletal disorders [26, 27]. However, case categorization in the setting of research or daily clinical routine needs to consider feasibility and the availability of resources. Hence, Dionne et al [28] proposed a minimal and optimal case definition for categorizing musculoskeletal diseases depending on its research purpose to promote balanced results. In contrast to the aforementioned studies, focusing on the preceding etiological aspect of musculoskeletal diseases, the sorting algorithm proposed in our study steps in to manage post-image acquisition of musculoskeletal diseases, and yet is in line with the aforementioned approach of Dionne et al since our proposed pre-sorting algorithm based on the anatomical entity provides a minimal still optimal tool for classifying radiographs in the assessment of musculoskeletal diseases. Since the expansion of radiological exams is most likely to generate large volumes of information and in order to establish a common hub to facilitate such large image datasets with potentially underlying musculoskeletal diseases, the data in our study were categorized according to their anatomical entity since most musculoskeletal diseases are, at least in part, classified according to the anatomical entity in which they are located [2].

To the best of our knowledge, no framework for curating and categorizing medical radiographs by musculoskeletal characteristics has yet been proposed. However, related problems have been addressed. In 2005, Lehmann et al [29] proposed automatic categorization of medical images into 80 classes, e.g., by imaging modality and biological system in the context of content-based image retrieval (CBIR). Uwimana et al [30] also demonstrated a content-based image retrieval model by establishing links between low-level features of images and high-level features of text codes. Gál et al [31] proposed a CBIR model with a multidisciplinary approach to solve the classification problem by combining image features, metadata, textual, and referential information. More recently, Guo et al [32] presented an interactive algorithm for dermatological image quantification that combines computation, visualization, and expert interaction. The most comparable study was proposed by Kart et al [33]: DeepMCAT, an unsupervised clustering approach also based on DeepCluster [8]. An end-to-end training automatically categorizes large-scale cardiac MR images into 13 classes without any annotation. The main differences with the method presented by Kart et al are that we integrated weak annotation to train the CNN according to our requirements and we aim to categorize X-ray images into 28 classes. However, we hypothesize that our approach generally can achieve high results due to the implementation of powerful state-of-the-art self-supervised methodology while keeping the demand of human interaction at a limit and being adaptable to other requirements with only minor adjustments.

We acknowledge that our study has several limitations. Firstly, the classifier did assume an input of images that relate to one of the (pre)defined radiographic classes. Images that would have been derived of a different image modality, such as ultrasound or cross-sectional imaging, and falsely merged to our SAM-X model, would not be detected as such but forced into one of the classes. However, this is a common issue with AI classification models and needs to be addressed in the future. Secondly, weak supervision is an approach of machine learning that uses imprecise (noisy) data for supervised learning usually by bypassing the time-consuming task of hand-labelling the whole dataset [34] for example through obtaining weak labels with clustering methods. Assuming that some images were “incorrectly” clustered even though the region of interested is present in the image (e.g., shoulder and clavicula or femur and knee), it is reasonable to consider multiple labels for a single image. To address this issue, we calculated a second accuracy score and considered the two predictions with the highest probability (as presented in the “Results” section). The respective scores for validation and testing reached 99.7% and 99.6% (in comparison to a single label 96.2 and 96.6). These numbers indicate that the model did not weakly label with respect to any incidental image features such as background or artefacts, but did indeed label according to similar anatomical features.

In conclusion, to facilitate the increasing amount of radiology examinations, accompanied by large image datasets, we propose a precise human-in-the-loop sorting algorithm for classifying radiographs in the assessment of musculoskeletal diseases according to the anatomical entity in which they are located. For dataset building, the algorithm proposes to be an efficient and time-saving tool in the setting of post-image acquisition.

## Abbreviations

AI: Artificial intelligence
CBIR: Content-based image retrieval
DL: Deep Learning
NMI: Normalized mutual information
PACS: Picture Archiving and Communication System

## Declarations

### Guarantor

The scientific guarantor of this publication is Jan Neumann.

### Statistics and biometry

No complex statistical methods were necessary for this paper.

### Methodology

• retrospective

• diagnostic or prognostic study/experimental

• performed at one institution
